# Ocrelizumab efficacy in subgroups of patients with relapsing multiple sclerosis

**DOI:** 10.1007/s00415-019-09248-6

**Published:** 2019-02-28

**Authors:** Benjamin Turner, Bruce A. C. Cree, Ludwig Kappos, Xavier Montalban, Caroline Papeix, Jerry S. Wolinsky, Regine Buffels, Damian Fiore, Hideki Garren, Jian Han, Stephen L. Hauser

**Affiliations:** 1 Department of Neurology, The Royal London Hospital, London, E1 1BB UK; 2 Weill Institute for Neurosciences, Department of Neurology, University of California, San Francisco, San Francisco, CA USA; 3 University Hospital Basel, University of Basel, Basel, Switzerland; 4 Vall d’Hebron University Hospital, Barcelona, Spain; 5 Division of Neurology, St Michael’s Hospital, University of Toronto, Toronto, Canada; 6 Pitié-Salpêtrière Hospital, Paris, France; 7 McGovern Medical School, The University of Texas Health Science Center at Houston (UTHealth), Houston, TX USA; 8 F. Hoffmann-La Roche Ltd, Basel, Switzerland; 9 Genentech, Inc., South San Francisco, CA USA

**Keywords:** Relapsing, Multiple sclerosis, Ocrelizumab, Subgroup, Interferon β-1a, Phase 3

## Abstract

**Objective:**

The efficacy and safety of ocrelizumab, versus interferon (IFN) β-1a, for the treatment of relapsing multiple sclerosis (RMS) from the identically designed OPERA I (NCT01247324) and OPERA II (NCT01412333) phase III studies has been reported; here we present subgroup analyses of efficacy endpoints from the pooled OPERA I and OPERA II populations.

**Methods:**

Patients with RMS were randomized to either ocrelizumab 600 mg administered by intravenous infusion every 24 weeks or subcutaneous IFN β-1a 44 µg three times per week throughout the 96-week treatment period. Relapse, disability, and MRI outcomes were analyzed for predefined and post hoc subgroups based on demographic and disease characteristics along with prior treatment using appropriate statistical tests to determine the treatment effect in subgroups and treatment-by-subgroup interactions.

**Results:**

The significant treatment benefit of ocrelizumab, versus IFN β-1a, observed in the overall OPERA I and OPERA II pooled populations was maintained across most subgroup strata for all endpoints, including annualized relapse rate, disability progression, and MRI outputs.

**Conclusions:**

The treatment effect of ocrelizumab versus IFN β-1a, measured by clinical and MRI outcomes, was maintained across most of the subgroups and strata of interest, and the pattern of treatment benefit across all subgroups was consistent with that from the pooled OPERA studies.

**Electronic supplementary material:**

The online version of this article (10.1007/s00415-019-09248-6) contains supplementary material, which is available to authorized users.

## Introduction

The efficacy and safety of ocrelizumab, a humanized CD20^+^ B-cell selective monoclonal antibody, versus high-dose, high-frequency interferon (IFN) β-1a in patients with relapsing multiple sclerosis (RMS), was demonstrated in the phase III OPERA I and OPERA II studies [1]. The identical study designs, comparable baseline demographics and disease characteristics of patients, and the evidence of no interaction between treatment and trial on the primary endpoint (annualized relapse rate [ARR]) between the OPERA I and OPERA II trials [1], permitted pooling of trial data.

Compared with IFN β-1a, treatment with ocrelizumab resulted in a 47% lower ARR (primary endpoint; *p* < 0.001) in the analysis of the pooled OPERA I and OPERA II intent-to-treat (ITT) population [2]. Similarly, treatment with ocrelizumab was associated with relatively lower rates of disability progression confirmed at 12 weeks (12W-CDP; 40%) and at 24 weeks (24W-CDP; 40%) in the prespecified pooled analysis of both studies. These data were further supported by a significantly greater suppression of development of new areas of inflammation (assessed by MRI of the brain with the use of gadolinium enhancement) and new or newly enlarged plaque formation (as measured by lesions on T2-weighted MRI) [1].

The current analyses were undertaken to understand if the treatment effects of ocrelizumab are consistent across subgroups of patients with different baseline characteristics; to describe the efficacy of ocrelizumab in patient subgroups relating to disability and clinical and MRI disease activity; and to describe the efficacy of ocrelizumab in both treatment-naïve patients and those previously treated with disease-modifying therapy (DMT).

## Methods

### Trial design and patients

The methodology of the identically designed phase III OPERA I (NCT01247324) and OPERA II (NCT01412333) studies has been reported previously [1]. Patients (aged 18–55 years, McDonald criteria [2010] diagnosis of MS [3], baseline Expanded Disability Status Scale [EDSS] score 0–5.5, ≥ 2 documented clinical relapses within the previous 2 years or one clinical relapse within the year before screening) were randomized (1:1) to treatment with either ocrelizumab 600 mg administered by intravenous infusion every 24 weeks, or subcutaneous IFN β-1a 44 µg three times per week throughout the 96-week treatment period. All patients provided written informed consent.

### Statistical analyses

Statistical approaches, including sample size calculations and analyses of primary and secondary endpoints, have been described previously [1].

Subgroup analyses, based on data from the 96-week double-blind treatment period, of the primary (ARR) and secondary endpoints were assessed in the ITT population (all randomized patients [ocrelizumab, *n* = 827; IFN β-1a, *n* = 829]); analyses of no evidence of disease activity (NEDA) were conducted in the modified ITT (mITT) population (ocrelizumab, *n* = 761; IFN β-1a, *n* = 759), where patients withdrawn from the trial for reasons other than efficacy failure or death and who had no evidence of clinical disease activity at the time of treatment discontinuation in the trial were excluded [1].

Prespecified subgroups were: study (OPERA I versus OPERA II), age (< 40 versus ≥ 40 years), sex, BMI (< 25 kg/m^2^ versus ≥ 25 kg/m^2^), region (USA versus rest of world), baseline EDSS score (< 4 versus ≥ 4), baseline gadolinium-enhancing T1 lesion status (0 versus ≥ 1), and pre-treated patients with active disease (pre-treated for ≥ 1 year with either ≥ 1 relapse in the year prior to randomization or ≥ 1 baseline T1 gadolinium-enhancing lesion) or highly active disease (≥ 1 relapse in the year prior to randomization and ≥ 9 T2 lesions or ≥ 1 T1 gadolinium-enhancing lesion at baseline). Additional post hoc subgroups, included because of their clinical relevance, consisted of: baseline EDSS score (< 2.5 versus ≥ 2.5 [for further evaluation of patients with lower disability at baseline]), prior DMT use within the 2 years prior to study inclusion (yes versus no), prior relapse (≤ 1 versus ≥ 2), and baseline normalized brain volume (≥ 1500 cm^3^ versus < 1500 cm^3^).

### Statistical analyses of endpoint by subgroup

Subgroup-level treatment comparisons were used to determine whether the treatment effects of ocrelizumab and IFN β-1a were the same within each subgroup level. Treatment-by-subgroup interaction tests were used to determine whether the treatment effect of ocrelizumab versus IFN β-1a was the same between the two levels of subgroup. *p* values < 0.05 from the treatment-by-subgroup interaction test indicate that the treatment effect of ocrelizumab versus IFN β-1a was not the same between the two levels of subgroup.

For ARR, both subgroup-level and treatment-by-subgroup interactions testing were performed using a negative binomial or quasi-Poisson model with the number of relapses as the response variable and log-transformed exposure time as the offset variable in both models. Factors included in subgroup-level tests were treatment, study, region, and baseline EDSS score (< 4.0 versus ≥ 4.0); additional factors in treatment-by-subgroup interaction testing were subgroup and treatment-by-subgroup interaction.

Disability progression, with 12- or 24-week confirmation, subgroup-level, and treatment-by-subgroup interactions testing were performed using Cox proportional hazard models with time to onset of disability progression as the response variable and treatment (ocrelizumab versus IFN β-1a) as a factor, and study, region and baseline EDSS score (< 4.0 versus ≥ 4.0) as adjustments in both models; additional factors in the treatment-by-subgroup interaction testing were subgroups and treatment-by-subgroup interaction.

For the MRI outcomes of T1 gadolinium-enhancing lesions and new/enlarging T2 lesions, subgroup-level and treatment-by-subgroup interactions testing were performed using a negative binomial or quasi-Poisson model with the number of lesions as the response variable, the log-transformed number of MRI scans as the offset variable, and baseline lesion count, treatment, study, region, and baseline EDSS score (< 4.0 versus ≥ 4.0) as factors in both models; additional factors in the treatment-by-subgroup interaction tests were subgroup and treatment-by-subgroup interaction. For change from baseline brain volume, subgroup and treatment-by-subgroup interaction testing used a mixed-effect model of repeated measures model (unstructured covariance matrix) with percentage change in brain volume as the dependent variable and baseline brain volume, treatment, study, region, baseline EDSS score (< 4.0 versus ≥ 4.0), week, baseline brain volume-by-week, and treatment-by-week as factors in both models; additional factors in the treatment-by-subgroup interaction tests were subgroup and treatment-by-week-by-subgroup.

Subgroup-level testing of NEDA or NEDA 24–96 (NEDA rebaselined at Week 24, which provides a representation of steady-state efficacy unconfounded by any initial disease activity carried over from baseline and recent pre-baseline disease state [4]) used the Cochran–Mantel–Haenszel test with treatment and NEDA status as the column/row factors and study, region, and baseline EDSS score (< 4.0 versus ≥ 4.0) as stratification factors. Treatment-by-subgroup interaction used the Breslow–Day test with treatment/NEDA status as the column/row factors and subgroup as the stratification factor.

For subgroup-level analyses, key covariates (i.e., study, region, or baseline EDSS < 4.0 versus ≥ 4.0) were not included as a main effect if the key covariate was used as the subgroup. If the subgroup was EDSS < 2.5 versus ≥ 2.5, then baseline EDSS < 4.0 versus ≥ 4.0 was not included as a main effect.

Analyses of patients who were pre-treated and had active or highly active disease were conducted in a similar way to the subgroup-level analyses described above, with the exception that no treatment-by-subgroup testing was conducted.

## Results

Patient disposition, demographic and disease characteristics, and safety findings from the individual OPERA I and OPERA II studies were reported previously [1]. Baseline demographic and disease characteristics between treatment groups in the pooled ITT population were generally comparable (Table 1), and characteristics within the mITT population were generally comparable to those within the ITT population (Supplementary Table S1).

**Table 1:** Baseline demographic and disease characteristics of the pooled OPERA I and OPERA II intent-to-treat population

Characteristic	IFN β-1a 44 µg (*N* = 829)	Ocrelizumab 600 mg (*N* = 827)
Age		
Years, mean (SD)	37.2 (9.2)	37.1 (9.2)
< 40 years, *n* (%)	484 (58.4)	496 (60.0)
≥ 40 years, *n* (%)	345 (41.6)	331 (40.0)
Female, *n* (%)	552 (66.6)	541 (65.4)
Body mass index		
kg/m^2^, mean (SD)	26.4 (6.2)	26.2 (5.8)
< 25 kg/m^2^, *n* (%)	413 (50.2)	406 (49.6)
≥ 25 kg/m^2^, *n* (%)	409 (49.8)	412 (50.4)
Time since MS symptom onset, years, mean (SD)	6.5 (6.1)	6.7 (6.2)
Time since RMS diagnosis, years, mean (SD)	3.9 (4.9)	4.0 (4.9)
No DMT in the 2 years before study inclusion, *n* (%)	606 (73.4)^a,b^	605 (73.3)^a,b^
EDSS score		
Mean (SD)	2.8 (1.3)^c^	2.8 (1.3)
< 2.5, *n* (%)	329 (39.7)^c^	310 (37.5)
≥ 2.5, *n* (%)	499 (60.3)^c^	517 (62.5)
< 4.0, *n* (%)	627 (75.7)^c^	629 (76.1)
≥ 4.0, *n* (%)	201 (24.3)^c^	198 (23.9)
Number of relapses, mean (SD)		
In the last year, mean (SD)	1.33 (0.69)^d^	1.32 (0.67)^d^
In the last 2 years, mean (SD)	1.76 (0.92)^d^	1.79 (0.91)^d^
≤ 1 relapse, *n* (%)	584 (70.6)^d^	585 (70.8)^d^
≥ 2 relapses, *n* (%)	243 (29.4)^d^	241 (29.2)^d^
MRI		
Patients with no T1 gadolinium-enhancing lesions, *n* (%)	495 (60.2)^e^	485 (59.3)^e^
Patients with ≥ 1 T1 gadolinium-enhancing lesion, *n* (%)	327 (39.8)^e^	333 (40.7)^e^
Number of T2 lesions, mean (SD)	51 (38)^f^	50 (39)^f^
T2 lesion volume, cm^3^, mean (SD)	10.18 (11.8)^f^	10.79 (14.1)^f^
Normalized brain volume, cm^3^, mean (SD)	1500 (89)^g^	1502 (88)^g^
Normalized brain volume < 1500 cm^3^, *n* (%)	398 (48.7)^g^	402 (49.0)^g^
Normalized brain volume ≥ 1500 cm^3^, *n* (%)	420 (51.3)^g^	418 (51.0)^g^

*DMT* disease-modifying therapy, *EDSS* Expanded Disability Status Scale, *IFN* interferon, *MRI* magnetic resonance imaging, *MS* multiple sclerosis, *OCR* ocrelizumab, *RMS* relapsing multiple sclerosis, *SD* standard deviation

^a^Data include patients who were untreated with any DMT in the 2 years before screening

^b^IFN β-1a *n* = 826, OCR *n* = 825

^c^IFN β-1a *n* = 828

^d^IFN β-1a *n* = 827, OCR *n* = 826

^e^IFN β-1a *n* = 822, OCR *n* = 818

^f^IFN β-1a *n* = 824, OCR *n* = 822

^g^IFN β-1a *n* = 818, OCR *n* = 820

### Subgroup analyses

#### Annualized relapse rate

**Fig. 1:**
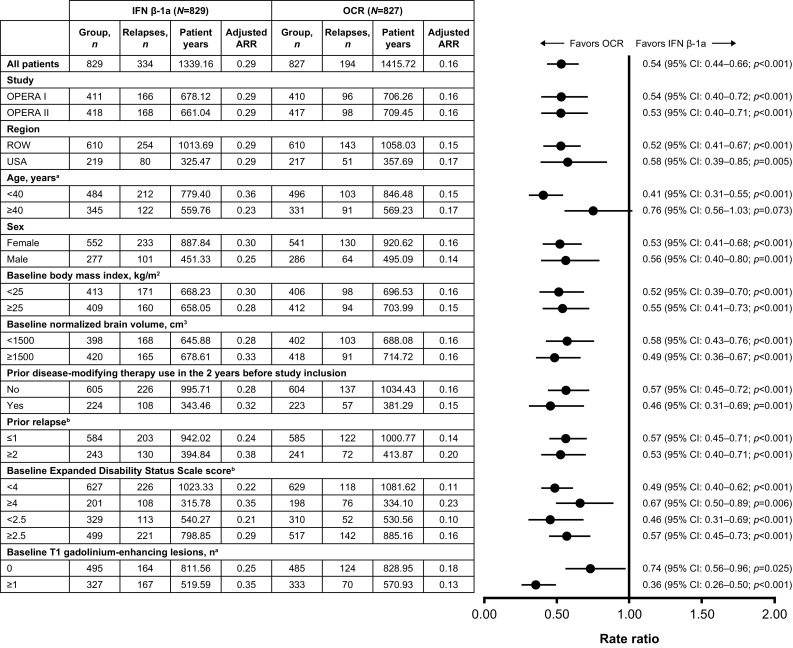
Annualized relapse rate by subgroup in the pooled OPERA I and OPERA II intent-to-treat population. Subgroup-level and treatment-by-subgroup interactions were assessed by the negative binomial method, or quasi-Poisson model if appropriate (where indicated). *ARR* annualized relapse rate, *CI* confidence interval, *IFN* interferon, *OCR* ocrelizumab, *ROW* rest of world. ^a^A significant treatment-by-subgroup interaction was observed for age (< 40 versus ≥ 40 years; *p* = 0.006) and for baseline T1 gadolinium-enhancing lesion status (0 versus ≥ 1; *p* < 0.001); *p* values < 0.05 from the treatment-by-subgroup interaction test indicate that the treatment effect of OCR versus IFN is not the same at each subgroup-level. ^b^Estimated by quasi-Poisson model

The significant reduction in ARR observed in the overall pooled analysis of the ITT population with ocrelizumab, relative to IFN β-1a (47% [*p* < 0.001]), was maintained across the majority of subgroup-levels, including study, region, age, sex, baseline BMI, prior DMT use and prior relapse, baseline EDSS, normalized brain volume, and gadolinium-enhancing T1 lesions; a numerical trend favoring ocrelizumab was observed for the specific subgroup strata of patients aged ≥ 40 years (Fig. 1).

Significant treatment-by-subgroup interactions for ARR were observed between patients aged < 40 versus ≥ 40 years (*p* = 0.006) and patients with or without baseline T1 gadolinium-enhancing lesions (*p* < 0.001). Treatment-by-subgroup interaction *p* values for all endpoints and subgroups are presented in Supplementary Table S2.

### Confirmed disability progression

**Fig. 2:**
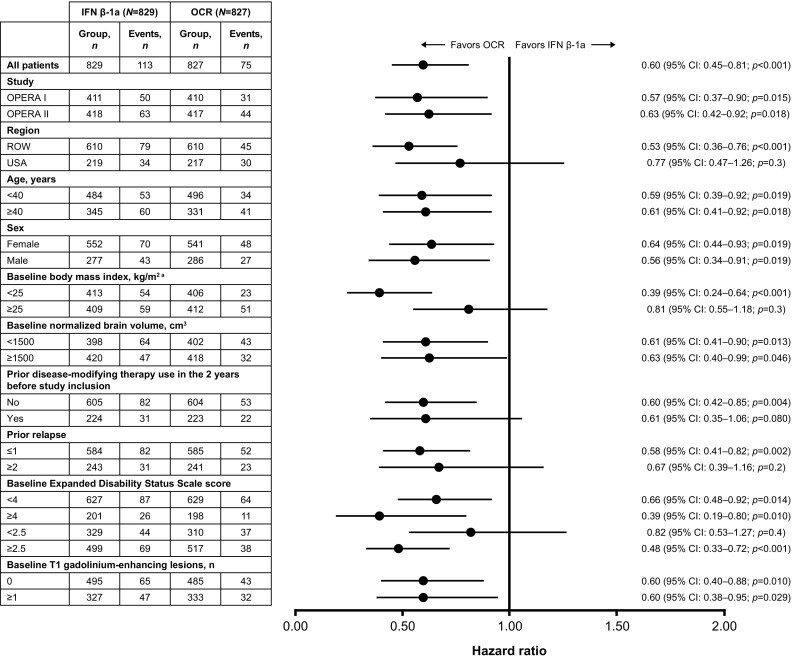
Disease progression confirmed at Week 12 by subgroup in the pooled OPERA I and OPERA II intent-to-treat population. Subgroup-level and treatment-by-subgroup interactions were assessed by the Cox proportional hazards method. *CI* confidence interval, *IFN* interferon, *OCR* ocrelizumab, *ROW* rest of world. ^a^A significant treatment-by-subgroup interaction was observed for BMI (< 25 kg/m^2^ versus ≥ 25 kg/m^2^; *p* = 0.026); *p* values < 0.05 from the treatment-by-subgroup interaction test indicate that the treatment effect of OCR versus IFN is not the same at each subgroup-level

The significant reductions in disease progression with 12-week confirmation (40% [*p* < 0.001]; Fig. 2) and 24-week confirmation (40% [*p* = 0.003]; Supplementary Fig. S1), relative to IFN β-1a, observed in the overall pooled analysis of the ITT population were maintained across most subgroup-levels.

For 12W-CDP, numerical trends favoring ocrelizumab were observed in the subgroup strata of patients: from the USA, who received prior DMT, with ≥ 2 prior relapses at baseline, with a baseline EDSS score < 2.5, or with a baseline BMI ≥ 25 kg/m^2^ (Fig. 2). The pattern of numerical trends favoring ocrelizumab for 24W-CDP was the same as for 12W-CDP with the addition of trends for males and for patients with a baseline normalized brain volume < 1500 cm^3^ (Supplementary Fig. S1).

Significant treatment-by-subgroup interactions were observed between patients with a baseline BMI < 25 kg/m^2^ versus ≥ 25 kg/m^2^ for both 12W-CDP (*p* = 0.026) and 24W-CDP (*p* = 0.016; Fig. 2).

### MRI outcomes

**Fig. 3:**
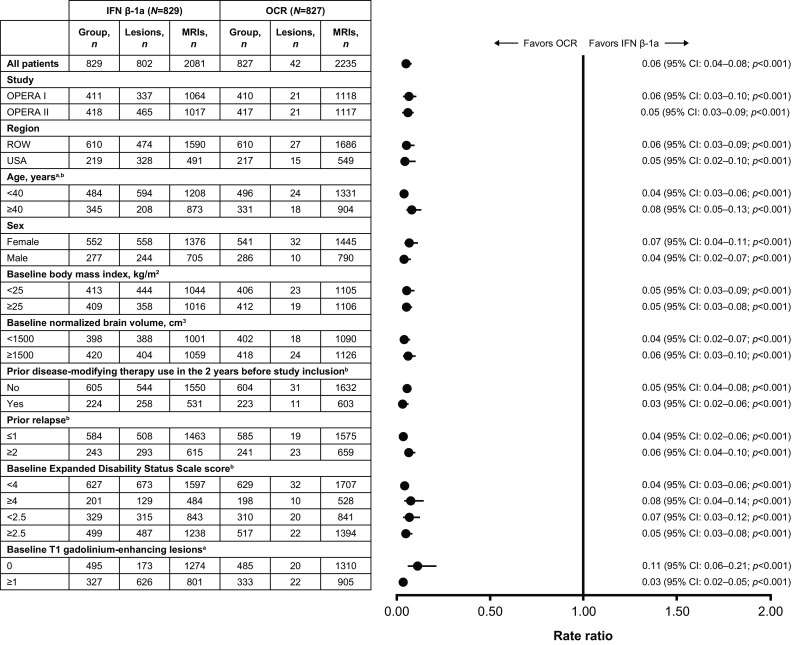
T1 gadolinium-enhancing lesions by subgroup in the pooled OPERA I and OPERA II intent-to-treat population. Subgroup-level and treatment-by-subgroup interactions were assessed by the negative binomial method, or quasi-Poisson model if appropriate (where indicated). *CI* confidence interval, *IFN* interferon, *MRI* magnetic resonance imaging, *OCR* ocrelizumab, *ROW* rest of world. ^a^A significant treatment-by-subgroup interaction was observed for age (< 40 versus ≥ 40 years; *p* = 0.030) and for baseline T1 gadolinium-enhancing lesion status (0 versus ≥ 1; *p* = 0.001); *p* values < 0.05 from the treatment-by-subgroup interaction test indicate that the treatment effect of OCR versus IFN is not the same at each subgroup-level. ^b^Estimated by quasi-Poisson model

**Fig. 4:**
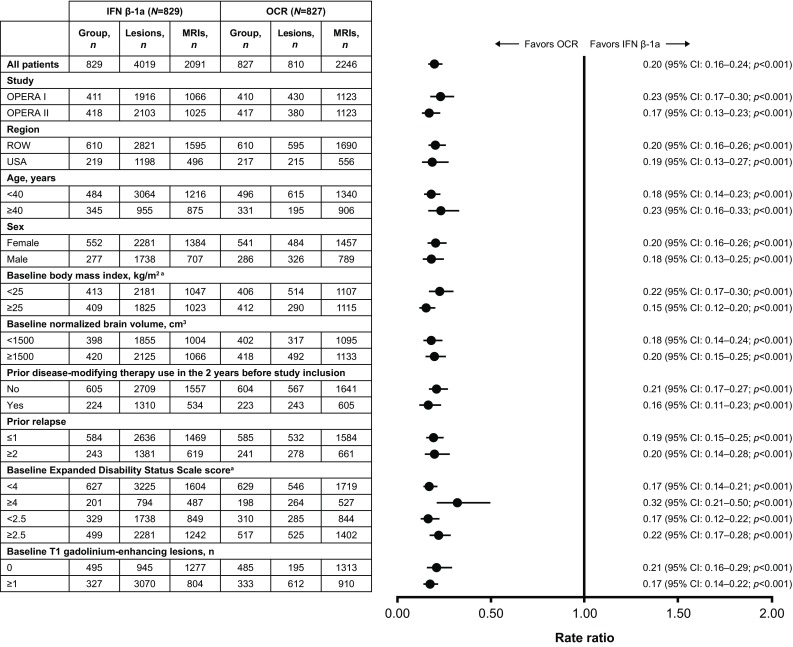
New/enlarging T2 lesions by subgroup in the pooled OPERA I and OPERA II intent-to-treat population. Subgroup-level and treatment-by-subgroup interactions were assessed by the negative binomial method, or quasi-Poisson model if appropriate (where indicated). *CI* confidence interval, *EDSS* Expanded Disability Status Scale, *IFN* interferon, *MRI* magnetic resonance imaging, *OCR* ocrelizumab, *ROW* rest of world. ^a^A significant treatment-by-subgroup interaction was observed for baseline BMI (< 25 versus ≥ 25 kg/m^2^; *p* = 0.043) and baseline EDSS score (< 4.0 versus ≥ 4.0; *p* = 0.007); *p* values < 0.05 from the treatment-by-subgroup interaction test indicate that the treatment effect of OCR versus IFN is not the same at each subgroup-level

The substantial reductions in both the average number of gadolinium-enhancing T1 lesions (94% [*p* < 0.001]; Fig. 3) and the average number of new or newly enlarged hyperintense T2 lesions per scan (80% [*p* < 0.001]; Fig. 4), of ocrelizumab relative to IFN β-1a, observed in the overall pooled analysis of the ITT population were maintained across all subgroup-levels (*p* < 0.001 for both endpoints and all comparisons).

For T1 gadolinium-enhancing lesions, treatment-by-subgroup interactions were observed between patients aged < 40 versus ≥ 40 years (*p* = 0.030) and patients with or without baseline T1 gadolinium-enhancing lesions (*p* = 0.001). With respect to new or newly enlarged hyperintense T2 lesions, treatment-by-subgroup interactions were observed between patients with a baseline EDSS score < 4.0 versus ≥ 4.0 (*p* = 0.007) and a baseline BMI < 25 kg/m^2^ versus ≥ 25 kg/m^2^ (*p* = 0.043).

**Fig. 5:**
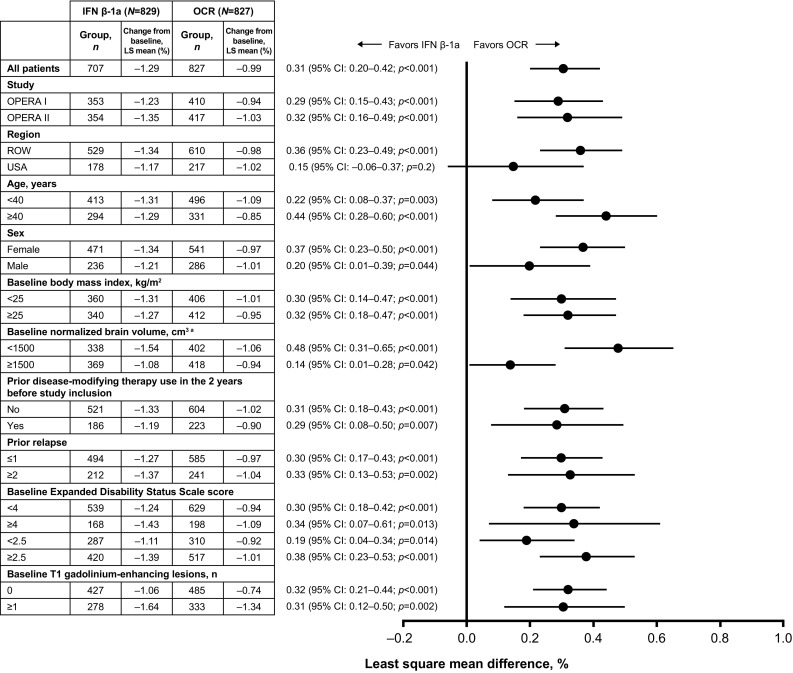
Change from baseline brain volume (%) by subgroup in the pooled OPERA I and OPERA II intent-to-treat population. Subgroup-level and treatment-by-subgroup interactions were assessed by the mixed-effect model of repeated measures model. *CI* confidence interval, *IFN* interferon, *LS* least square, *OCR* ocrelizumab, *ROW* rest of world. ^a^A significant treatment-by-subgroup interaction was observed for normalized baseline brain volume (< 1500 cm^3^ and ≥ 1500 cm^3^; *p* = 0.002); *p* values < 0.05 from the treatment-by-subgroup interaction test indicate that the treatment effect of OCR versus IFN is not the same at each subgroup-level

The benefit in change from baseline brain volume observed in the overall pooled analysis of the ITT population with ocrelizumab, relative to IFN β-1a (least square mean difference, 0.31% [*p* < 0.001]; Fig. 5), was maintained across the majority of subgroup-levels; a numerical trend favoring ocrelizumab was observed in patients from the USA. A treatment-by-subgroup interaction was observed between patients with a normalized baseline brain volume < 1500 cm^3^ and ≥ 1500 cm^3^ (*p* = 0.002).

### No evidence of disease activity

**Fig. 6:**
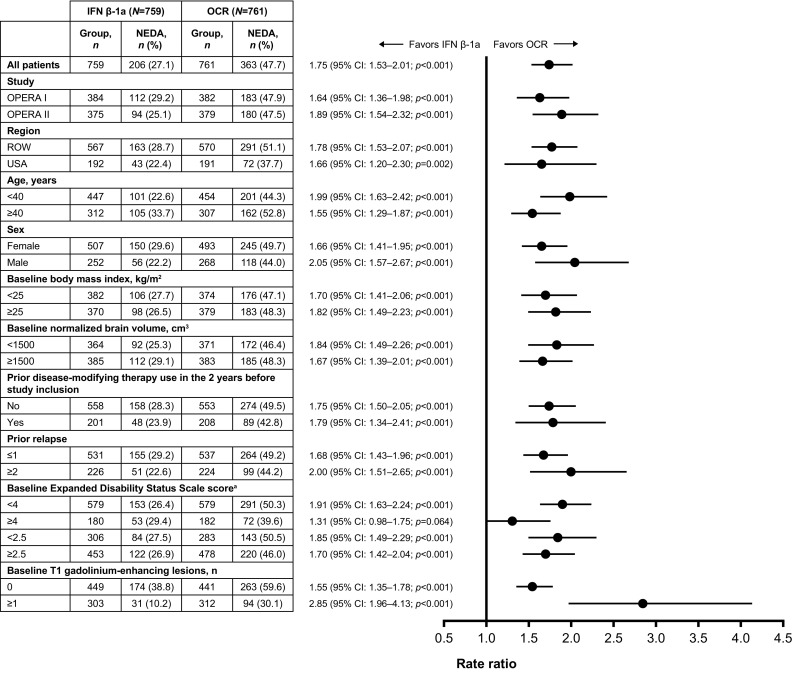
Subgroup analyses of the proportion of patients with NEDA in the pooled OPERA I and OPERA II mITT population. Subgroup-level testing used the Cochran–Mantel–Haenszel test and treatment-by-subgroup interactions were assessed by the Breslow–Day test. *CI* confidence interval, *EDSS* Expanded Disability Status Scale, *IFN* interferon, *mITT* modified intent-to-treat, *NEDA* no evidence of disease activity, *OCR* ocrelizumab, *ROW* rest of world. ^a^A significant treatment-by-subgroup interaction was observed for baseline EDSS score (< 4.0 versus ≥ 4.0; *p* = 0.022); *p* values < 0.05 from the treatment-by-subgroup interaction test indicate that the treatment effect of OCR versus IFN is not the same at each subgroup-level

The increase in the proportion of patients with NEDA in the overall pooled mITT population with ocrelizumab (75% [*p* < 0.001]), relative to IFN β-1a, was maintained across most subgroup-levels (Fig. 6); a numerical trend favoring ocrelizumab was observed in patients with a baseline EDSS score ≥ 4.0. A treatment-by-subgroup interaction was observed between patients with a baseline EDSS score < 4.0 and ≥ 4.0 (*p* = 0.022).

Similarly, when NEDA was rebaselined to Week 24, the improvement in the proportion of patients with NEDA 24–96 seen in the overall pooled mITT population with ocrelizumab (72% [*p* < 0.001]), relative to IFN β-1a, was maintained across all subgroup-levels (Supplementary Fig. S2). Treatment-by-subgroup interactions were observed between patients with a baseline EDSS score < 4.0 and ≥ 4.0 (*p* = 0.008), patients < 40 and ≥ 40 years old (*p* < 0.001), and those with or without T1 gadolinium-enhancing lesions at baseline (*p* < 0.001).

### Efficacy in pre-treated patients with active or highly active disease

A total of 301 patients were defined as pre-treated with active disease (ocrelizumab, *n* = 153; IFN β-1a, *n* = 148) and 283 patients were defined as pre-treated with highly active disease (ocrelizumab, *n* = 143; IFN β-1a, *n* = 140). Consistent with the reduction in ARR in the overall ITT pooled population (47%; *p* < 0.001), ocrelizumab, relative to IFN β-1a, reduced the ARR in patients who were pre-treated with active disease by 65% (*p* < 0.001) and pre-treated with highly active disease by 68% (*p* < 0.001).

Similar benefits in patients who were pre-treated with active disease or pre-treated with highly active disease were observed for 12W-CDP (54% relative reduction [*p* = 0.032] or a 53% relative reduction [*p* = 0.035], respectively) compared with a 40% reduction (*p* < 0.001) in the overall ITT pooled population; 24W-CDP (51% relative reduction [*p* = 0.075] or a 50% relative reduction [*p* = 0.082], respectively) compared with a 40% reduction (*p* = 0.003) in the overall ITT pooled population; T1 gadolinium-enhancing lesions (98% relative reduction [*p* < 0.001 in both subgroups]) compared with a 94% reduction (*p* < 0.001) in the overall ITT pooled population; and new or enlarging T2 lesions (80% relative reduction [*p* < 0.001 in both subgroups]) compared with an 80% reduction (*p* < 0.001) in the overall ITT pooled population.

For patients who were pre-treated with either active or highly active disease, the statistically significant treatment benefits of ocrelizumab relative to IFN β-1a were demonstrated for all endpoints except for 24W-CDP, which comprised a relatively smaller number of patients.

## Discussion

In the OPERA I and OPERA II studies, ocrelizumab, relative to IFN β-1a, was associated with significantly better outcomes in terms of ARR, disability progression, suppression of new inflammatory lesions in the brain (T1 gadolinium-enhancing lesions), and new or newly enlarged plaque formation (new/enlarging T2 lesions) [1]. The subgroup analyses presented here demonstrate that the significant treatment benefits of ocrelizumab, relative to IFN β-1a, are maintained across most baseline-, clinically, and radiologically defined subgroups, although the magnitude of the treatment benefit of ocrelizumab over IFN β-1a may be greater in one of the two levels of subgroup. Treatment-by-subgroup interactions were observed for some endpoints over particular subgroups; however, all showed a benefit (significant or numerical) of ocrelizumab over IFN β-1a at each of the two levels of subgroup.

OPERA I and OPERA II were not powered to detect statistically significant effects for all subgroup analyses, including those predefined and with pre-planned pooling (12W-CDP or 24W-CDP), or to test for heterogeneity between subgroups. Additionally, post hoc analyses of subgroups can be difficult to interpret. Within this analysis most outcomes and subgroups were predefined; only NEDA 24–96 and brain volume change from baseline for outcomes, and the subgroups baseline EDSS score < 2.5 versus ≥ 2.5, prior DMT use yes versus no, those with ≤ 1 or ≥ 2 prior relapses, and those with a baseline normalized brain volume of < 1500 cm^3^ or ≥ 1500 cm^3^, were post hoc considerations.

Consequently, given the relatively smaller numbers of subjects in some of the subgroups, findings in these subgroups should be interpreted with caution. Further studies with an expanded data set with improved assessment techniques would be required to address these points.

For ARR, gadolinium-enhancing T1 lesions, and NEDA rebaselined at Week 24, significant subgroup interactions suggested that patients who were younger and those with indicators of active inflammatory disease appeared to gain a greater treatment benefit with ocrelizumab, relative to IFN β-1a, than patients who were older and those without markers of active inflammatory disease. These findings are consistent with treatment benefit of other DMTs, which has previously been suggested to be higher in younger than in older patients on the basis of higher disease activity in younger populations with MS [5]. Subgroup analyses performed for other DMTs using either placebo or an active comparator, e.g., fingolimod [6, 7], cladribine [8], teriflunomide [9], dimethyl fumarate [10–12], alemtuzumab [13], and natalizumab [14, 15], also found that efficacy was highest in younger patients with more active disease.

Consistent effects across endpoints were seen for other subgroups of interest. There were no significant sex-based subgroup interactions and the same positive trends of treatment benefit with ocrelizumab were reported in female and male patients. The treatment benefit of ocrelizumab, relative to IFN β-1a, was comparable across endpoints in patients with active or highly active disease who were pre-treated with DMT, while prior treatment per se did not impact the magnitude of the beneficial effect of ocrelizumab. Across other endpoints, the magnitude of ocrelizumab’s treatment benefit on brain atrophy was greater in patients with lower baseline brain volume, which may be related to differential rates of atrophy in the patient subgroups defined by brain volume thresholds. For NEDA (Weeks 0–96) and new or enlarging T2 lesion endpoints, the treatment benefit of ocrelizumab was greater in patients with lower measures of disability at baseline. For both 12W-CDP and 24W-CDP, significant treatment-by-subgroup interactions for BMI indicate that the magnitude of ocrelizumab treatment benefit is greater in lighter patients versus heavier, consistent with lower ocrelizumab exposure in heavier patients (BMI > 25 kg/m^2^ versus < 25 kg/m^2^). Analyses have shown that patients with a higher ocrelizumab exposure had a greater benefit on 12W-CDP and 24W-CDP. Therefore, it might be possible that in the BMI subgroups the ocrelizumab exposure had an influence on confirmed disability progression results. However, a contradictory significant treatment-by-subgroup interaction between BMI strata was observed (*p* = 0.043) for the endpoint of new or enlarging T2 lesion endpoints, indicating a higher magnitude of ocrelizumab treatment benefit in heavier versus lighter patients; however, this finding requires further study before clinical meaningfulness can be confirmed. Subgroup analyses of other studies with MS DMTs have demonstrated an interaction between age and disability progression, with lower levels of progression in younger patients; this was not observed in the current study.

In conclusion, the treatment benefit of ocrelizumab versus IFN β-1a, measured by clinical and MRI outcomes, was maintained across almost all the subgroups and strata of interest, and the pattern of treatment benefit across all subgroups was consistent with that from the pooled OPERA studies. The results show that RMS patients with a wide range of demographic and clinical characteristics benefit more from treatment with ocrelizumab than from IFN β-1a.

### Data sharing statement

Qualified researchers may request access to individual patient-level data through the clinical study data request platform (http://www.clinicalstudydatarequest.com). Further details on Roche’s criteria for eligible studies are available here: https://clinicalstudydatarequest.com/Study-Sponsors/Study-Sponsors-Roche.aspx. For further details on Roche’s Global Policy on the Sharing of Clinical Information and how to request access to related clinical study documents, see: https://www.roche.com/research_and_development/who_we_are_how_we_work/clinical_trials/our_commitment_to_data_sharing.htm.

## Electronic supplementary material

Below is the link to the electronic supplementary material.

Supplementary material 1 (PDF 801 KB)
