# Supplementary material for: Ocrelizumab efficacy in subgroups of patients with relapsing multiple sclerosis
Source: J Neurol. 2019 Feb 28;266(5):1182–93. doi: 10.1007/s00415-019-09248-6 (PMC6469695; doi:10.1007/s00415-019-09248-6)
Supplement: Supplementary file 1 — Supplementary material 1 (PDF 801 KB) [file 415_2019_9248_MOESM1_ESM.pdf]

Supplementary Figures

Supplementary Fig. S1 Subgroup analyses of disease progression confirmed at Week 24 by subgroup in the pooled OPERA I and OPERA II intent-to-treat population

	IFN β-1a (*N*=829)		OCR (*N*=827)	
	Group, *n*	Events, *n*	Group, *n*	Events, *n*
**All patients**	829	87	827	57
**Study**				
OPERA I	411	39	410	24
OPERA II	418	48	417	33
**Region**				
ROW	610	63	610	36
USA	219	24	217	21
**Age, years**				
<40	484	42	496	27
≥40	345	45	331	30
**Sex**				
Female	552	55	541	33
Male	277	32	286	24
**Baseline body mass index, kg/m^{2}[a]**				
<25	413	47	406	18
≥25	409	40	412	38
**Baseline normalized brain volume, cm^{3}**				
<1500	398	46	402	34
≥1500	420	39	418	23
**Prior disease-modifying therapy use in the 2 years before study inclusion**				
No	605	64	604	39
Yes	224	23	223	18
**Prior relapse**				
≤1	584	60	585	37
≥2	243	27	241	20
**Baseline Expanded Disability Status Scale score**				
<4	627	66	629	47
≥4	201	21	198	10
<2.5	329	33	310	24
≥2.5	499	54	517	33
**Baseline T1 gadolinium-enhancing lesions, n**				
0	495	47	485	31
≥1	327	40	333	26

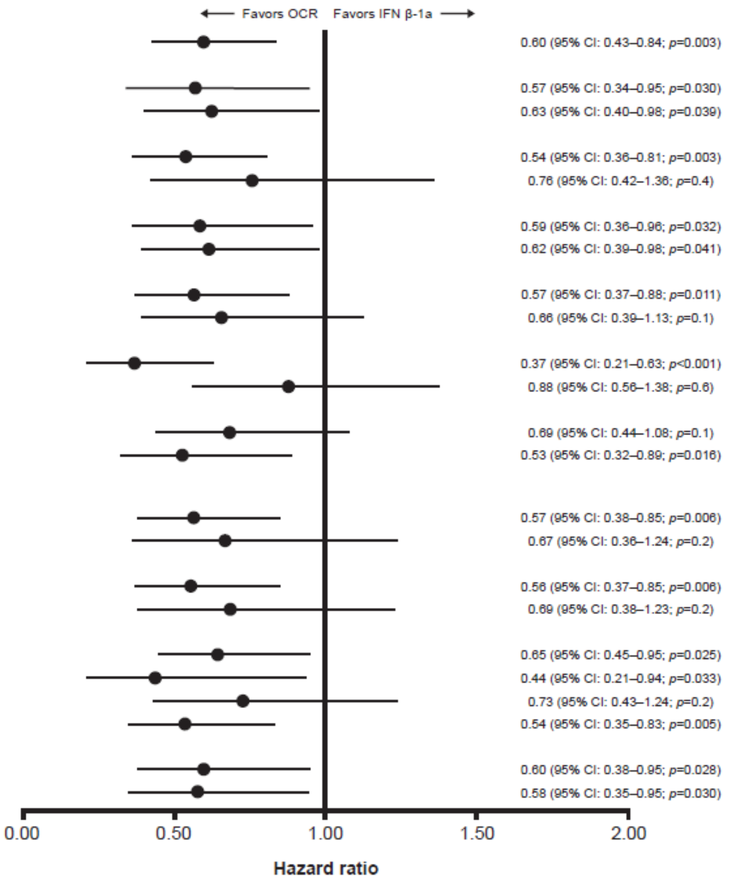

Subgroup level and treatment-by-subgroup interactions were assessed by the Cox proportional hazards method. *CI* confidence interval, *IFN* interferon, *OCR* ocrelizumab, *ROW* rest of world. [a]A significant treatment-by-subgroup interaction was observed for BMI (<25 years versus ≥25 kg/m^2; p=0.016); p-values <0.05 from the treatment-by-subgroup interaction test indicate that the treatment effect of OCR versus IFN is not the same at each subgroup level

Supplementary Fig. S2 Subgroup analyses of the proportion of patients with NEDA rebaselined at Week 24 (NEDA24–96) in the pooled OPERA I and OPERA II mITT population

	IFN β-1a (N=759)		OCR (N=761)		
	Group, n	NEDA, n (%)	Group, n	NEDA, n (%)	
**All patients**	706	296 (41.9)	745	538 (72.2)	1.72 (95% CI: 1.56–1.90; p<0.001)
**Study**					
OPERA I	360	158 (43.9)	375	270 (72.0)	1.64 (95% CI: 1.44–1.87; p<0.001)
OPERA II	346	138 (39.9)	370	268 (72.4)	1.82 (95% CI: 1.57–2.10; p<0.001)
**Region**					
ROW	538	237 (44.1)	561	420 (74.9)	1.70 (95% CI: 1.53–1.90; p<0.001)
USA	168	59 (35.1)	184	118 (64.1)	1.79 (95% CI: 1.42–2.26; p<0.001)
**Age, years[a]**					
<40	413	149 (36.1)	444	328 (73.9)	2.09 (95% CI: 1.82–2.41; p<0.001)
≥40	293	147 (50.2)	301	210 (69.8)	1.36 (95% CI: 1.19–1.56; p<0.001)
**Sex**					
Female	469	209 (44.6)	485	349 (72.0)	1.60 (95% CI: 1.42–1.79; p<0.001)
Male	237	87 (36.7)	260	189 (72.7)	2.04 (95% CI: 1.70–2.46; p<0.001)
**Baseline body mass index, kg/m²**					
<25	356	151 (42.4)	367	267 (72.8)	1.73 (95% CI: 1.52–1.98; p<0.001)
≥25	343	140 (40.8)	370	265 (71.6)	1.75 (95% CI: 1.51–2.02; p<0.001)
**Baseline normalized brain volume, cm³**					
<1500	343	139 (40.5)	360	250 (69.4)	1.71 (95% CI: 1.48–1.97; p<0.001)
≥1500	356	154 (43.3)	378	281 (74.3)	1.73 (95% CI: 1.52–1.98; p<0.001)
**Prior disease-modifying therapy use in the 2 years before study inclusion**					
No	527	232 (44.0)	544	393 (72.2)	1.64 (95% CI: 1.47–1.83; p<0.001)
Yes	179	64 (35.8)	201	145 (72.1)	2.02 (95% CI: 1.63–2.50; p<0.001)
**Prior relapse**					
≤1	495	213 (43.0)	528	387 (73.3)	1.70 (95% CI: 1.52–1.91; p<0.001)
≥2	210	83 (39.5)	217	151 (69.6)	1.77 (95% CI: 1.47–2.14; p<0.001)
**Baseline Expanded Disability Status Scale score[a]**					
<4	539	231 (42.9)	567	434 (76.5)	1.79 (95% CI: 1.61–2.00; p<0.001)
≥4	167	65 (38.9)	178	104 (58.4)	1.47 (95% CI: 1.18–1.85; p<0.001)
<2.5	285	130 (45.6)	279	217 (77.8)	1.71 (95% CI: 1.49–1.98; p<0.001)
≥2.5	421	166 (39.4)	466	321 (68.9)	1.75 (95% CI: 1.53–2.00; p<0.001)
**Baseline T1 gadolinium-enhancing lesions[a]**					
0	423	218 (51.5)	434	309 (71.2)	1.38 (95% CI: 1.24–1.54; p<0.001)
≥1	279	77 (27.6)	303	221 (72.9)	2.60 (95% CI: 2.13–3.17; p<0.001)

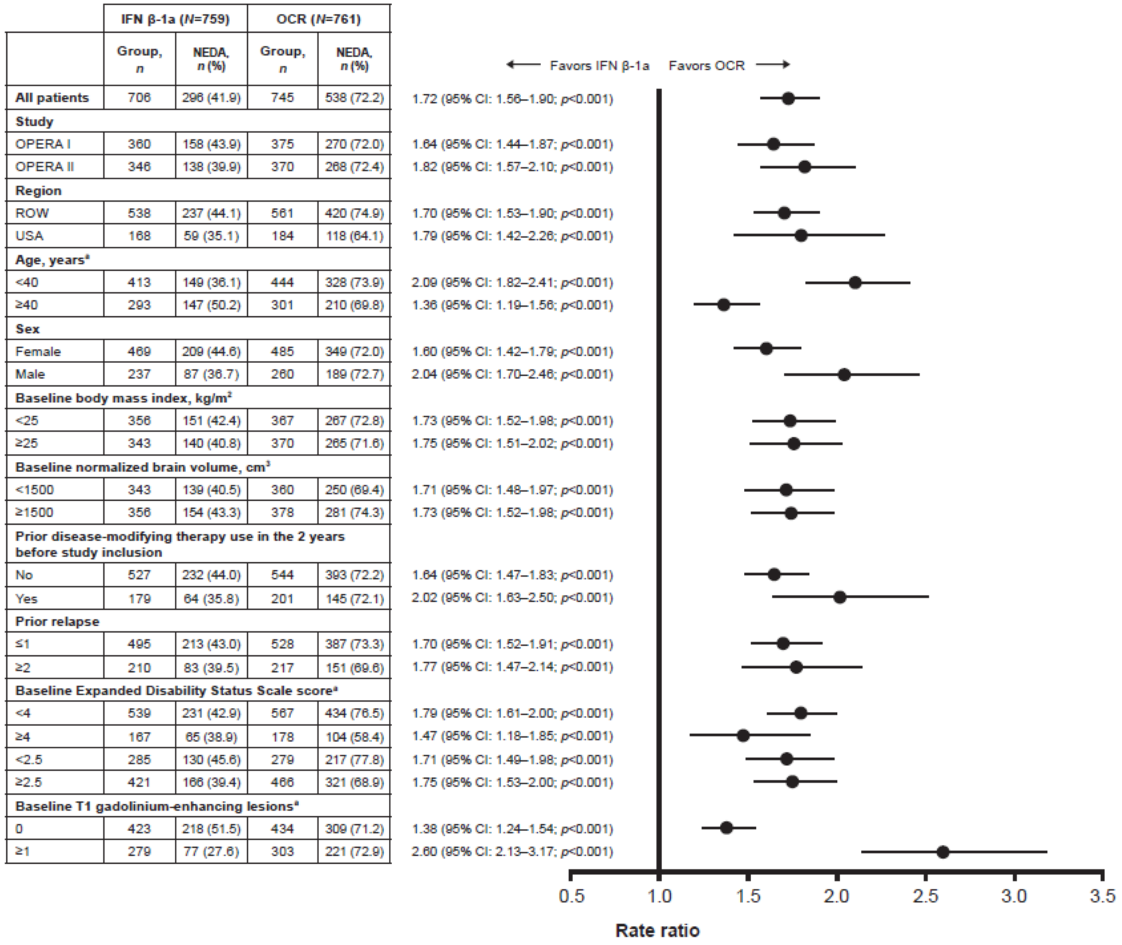

Subgroup level testing used the Cochran–Mantel–Haenszel test and treatment-by-subgroup interactions were assessed by the Breslow–Day test. *CI* confidence interval, *EDSS* Expanded Disability Status Scale, *IFN* interferon, *mITT* modified intent-to-treat, *NEDA* no evidence of disease activity, *OCR* ocrelizumab, *ROW* rest of world. [a]A significant treatment-by-subgroup interaction was observed for baseline EDSS score (<4.0 versus ≥4.0; p=0.008), age (<40 versus ≥40 years; p<0.001), and baseline T1 gadolinium-enhancing lesion status (0 versus ≥1; p<0.001); p-values <0.05 from the treatment-by-subgroup interaction test indicate that the treatment effect of OCR versus IFN is not the same at each subgroup level

Supplementary Tables

Supplementary Table S1 Baseline demographics and disease characteristics (pooled OPERA I and OPERA II mITT patient population)

**Characteristic**	**IFN β-1a 44 μg (*N*=759)**	**Ocrelizumab 600 mg (*N*=761)**
**Age**		
Years, mean (SD)	37.1 (9.2)	37.2 (9.1)
<40 years, n (%)	447 (58.9)	454 (59.7)
≥40 years, n (%)	312 (41.1)	307 (40.3)
**Female, n (%)**	507 (66.8)	493 (64.8)
**Body mass index**		
$Kg/m^{2,}$ mean (SD)	26.3 (6.1)	26.2 (5.8)
<25 kg/m^2, n (%)	382 (50.8%)	374 (49.7%)
≥25 kg/m^2, n (%)	370 (49.2%)	379 (50.3%)
**Time since MS symptom onset, mean (SD), years**	6.4 (6.1)	6.6 (6.1)
**Time since RMS diagnosis, mean (SD), years**	3.9 (4.8)	3.9 (4.9)
**No DMT in the 2 years before study inclusion, n (%)**	558 (73.5)	553 (72.7)
**EDSS score**		
Mean (SD)	2.8 (1.3)	2.8 (1.3)
<2.5, n (%)	306 (40.3)	283 (37.2)
≥2.5, n (%)	453 (59.7)	478 (62.8)

<4.0, n (%)	579 (76.3)	579 (76.1)
≥4.0, n (%)	180 (23.7)	182 (23.9)
**Number of relapses**		
In the last year, mean (SD)	1.3 (0.7)[a]	1.3 (0.7)
In the last 2 years, mean (SD)	1.8 (0.9)[a]	1.8 (0.9)[a]
≤1 relapse in the last year, n (%)	531 (70)[a]	537 (70.6)[a]
≥2 relapses in the last year, n (%)	226 (29.8)[a]	224 (29.4)
**MRI**		
Patients with no T1 gadolinium-enhancing lesions, n (%)	449 (59.7)	441 (58.6)
Patients with ≥1 T1 gadolinium-enhancing lesion, n (%)	303 (40.3)	312 (41.4)
Number of T2 hyperintense lesions, mean (SD)	51.0 (37.5)[b]	50.0 (38.7)[d]
T2 hyperintense lesion volume, cm^3, median (range)	6.2 (0–76.1)[b]	5.4 (0–96.0)[d]
Normalized brain volume, cm^3, mean (SD)	1,499.8 (88.3)[c]	1,501.7 (88.1)[b]
Normalized brain volume <1500 cm^3, n (%)	364 (48.6%)	371 (49.2%)
Normalized brain volume ≥1500 cm^3, n (%)	385 (51.4%)	383 (50.8%)

DMT disease-modifying therapy, *EDSS* Expanded Disability Status Scale, *IFN* interferon, *mITT* modified intent-to-treat, *MRI* magnetic resonance imaging, *MS* multiple sclerosis, *RMS* relapsing multiple sclerosis, *SD* standard deviation

[a]*n*=757. [b]*n*=754. [c]*n*=749. [d]*n*=75

Supplementary Table S2 Endpoint and *p*-values from treatment-by-subgroup interaction tests

	Endpoint *p*-value[a]							
**Subgroup**	**ARR**	**12W-CDP**	**24W-CDP**	**New / enlarging T2 lesion**	**T1 gadolinium-enhancing lesion**	**Change from baseline brain volume**	**NEDA**	**NEDA (Weeks 24–96)**
Study (OPERA I vs II)	0.9538	0.7664	0.8072	0.1020	0.8613	0.7464	0.3821	0.4055
Region (ROW vs USA)	0.7533	0.2059	0.3212	0.5387	0.6361	0.1028	0.4212	0.5994
Age (<40 vs ≥40)	0.0055	0.8675	0.8454	0.2339	0.0302	0.0608	0.3397	0.0006
Sex (female vs male)	0.7323	0.6031	0.6881	0.8494	0.1352	0.1412	0.5034	0.1261
BMI, kg/m^2 (<25 vs ≥25)	0.8329	0.0257	0.0163	0.0432	0.8096	0.8460	0.6068	0.9662
Prior DMT (yes vs no)	0.3824	0.8685	0.5977	0.1735	0.2287	0.9277	0.8671	0.1844
Prior relapse (≤1 vs ≥2)	0.7087	0.6292	0.5817	0.9661	0.0567	0.8798	0.5480	0.8786
Baseline EDSS								

(<4.0 vs ≥4.0)	0.1103	0.2324	0.4127	0.0068	0.1996	0.7496	0.0222	0.0079
(<2.5 vs ≥2.5)	0.3474	0.0932	0.4190	0.1655	0.3446	0.0866	0.4909	0.3820
Baseline T1 gadolinium-enhancing lesion (0 vs ≥1)	0.0009	0.9666	0.8945	0.3196	0.0012	0.9128	0.0673	<0.0001
Baseline normalized brain volume, cm^3 (<1500 vs ≥1500)	0.5053	0.9139	0.4700	0.5802	0.3766	0.0023	0.6008	0.5619

12W-CDP disability progression confirmed at 12 weeks, *24W-CDP* disability progression confirmed at 24 weeks, *ARR* annualized relapse rate, *DMT* disease-modifying therapy, *EDSS* Expanded Disability Status Scale, *IFN* interferon, *NEDA* no evidence of disease activity, *OCR* ocrelizumab, *ROW* rest of world

[a]*P*-values <0.05 from the treatment-by-subgroup interaction test indicate that the treatment effect of OCR versus IFN is not the same at each subgroup level
